# Clinical Significance of Hypokalemia in Blunt Liver Trauma: Preliminary Findings in a Retrospective Cohort Study of 164 Patients

**DOI:** 10.3390/jcm14113835

**Published:** 2025-05-29

**Authors:** Gioia Brachini, Giulia Duranti, Simona Meneghini, Marco La Torre, Eleonora Cianci, Pierfrancesco Lapolla, Luigi Simonelli, Emilio Gentile Warschauer, Roberto Cirocchi, Andrea Mingoli, Bruno Cirillo

**Affiliations:** 1 Department of Surgery, Sapienza University of Rome, 00185 Rome, Italy; gioia.brachini@uniroma1.it (G.B.); giuliaduranti95@gmail.com (G.D.); eleonoracianci6@gmail.com (E.C.); luigi.simonelli@policlinicoumberto1.it (L.S.); andrea.mingoli@uniroma1.it (A.M.); bruno.cirillo@uniroma1.it (B.C.); 2 Salvator Mundi International Hospital, UPMC, University of Pittsburgh Medical College, 00152 Rome, Italy; dott.marcolatorre@gmail.com; 3 Nuffield Department of Surgical Sciences, University of Oxford, Oxford OX1 2JD, UK; piefrancesco.lapolla@nds.ox.ac.uk; 4 Department of Surgery, Ospedale S. Pietro Fatebenefratelli, 00189 Rome, Italy; 5 Department of Surgery, General Surgery, University of Perugia, 06123 Perugia, Italy; roberto.cirocchi@unipg.it

**Keywords:** hypokalemia, blunt liver trauma, trauma biomarkers, potassium, outcome prediction

## Abstract

**Background:** The clinical significance of serum potassium levels at admission in patients with blunt hepatic trauma remains insufficiently defined. This study aimed to evaluate the prevalence and prognostic value of admission hypokalemia in this patient population. **Methods:** We conducted a retrospective analysis of 164 patients with radiologically confirmed blunt liver trauma admitted between 2016 and 2023. Preoperative, intraoperative, and postoperative data were collected to assess the association between serum potassium levels and trauma severity (AAST grade—American Association for the Surgery of Trauma, ISS—Injury Severity Score), in-hospital morbidity, mortality, and length of stay. Univariate and multivariate analyses were performed, including checks for normality and multicollinearity. **Results:** Serum potassium levels showed a significant positive correlation with age (*p* = 0.0064), and an inverse correlation with liver injury severity (AAST grade; *p* = 0.01). Lower potassium levels were associated with longer hospital stays (*p* = 0.0459) and higher morbidity (*p* = 0.022). In multivariate analysis, only age (*p* = 0.036) and AAST grade (*p* = 0.014) were independent predictors of serum potassium concentration. Potassium levels were not independently associated with mortality. **Conclusions:** Admission hypokalemia is a common finding in blunt liver trauma and correlates with injury severity and adverse clinical outcomes. Potassium concentration may serve as a readily available, low-cost biomarker for early risk stratification in these patients. Further prospective studies are warranted to confirm its prognostic utility.

## 1. Introduction

A significant decrease in serum potassium concentration is frequently observed following traumatic injury, particularly in patients with severe or multiple injuries. Although cell lysis and tissue damage would theoretically result in hyperkalemia due to the release of intracellular potassium, clinical and experimental studies consistently demonstrate that hypokalemia is more common in the early post-traumatic phase [1,2].

This paradoxical hypokalemia is primarily attributed to the intense neurohumoral response triggered by trauma. The acute release of catecholamines—especially epinephrine and norepinephrine—plays a central role in modulating potassium homeostasis through two main mechanisms:

1. β_2_-adrenergic receptor activation: Catecholamines stimulate the Na^+^/K^+^-ATPase pump via β_2_-adrenergic receptors, promoting intracellular potassium uptake and reducing extracellular concentrations [3].
2. Insulin-mediated uptake: The catecholaminergic response also induces hyperglycemia, leading to secondary insulin release. Insulin further activates the Na^+^/K^+^-ATPase, enhancing potassium influx into cells [4,5].

These combined mechanisms lower serum potassium levels, effectively masking the hyperkalemia expected from cellular damage. The magnitude and persistence of this electrolyte shift appear to correlate with the extent of tissue injury and adrenergic activation.

Brown et al. demonstrated that patients with American Association for the Surgery of Trauma (AAST) grade III–V liver injuries developed significant and persistent hypokalemia for up to seven days despite appropriate fluid and electrolyte support [6]. The liver’s dense sympathetic innervation and glycogen stores may contribute to this effect, making it particularly sensitive to catecholamine-induced shifts in potassium.

Similar trends have been observed in other trauma contexts, including traumatic brain and thoracic injuries, where early hypokalemia was associated with worse clinical outcomes [7,8]. Beyond its pathophysiological implications, trauma-related hypokalemia may have direct clinical consequences by contributing to arrhythmias, hemodynamic instability, and impaired organ perfusion [9].

To date, no large-scale studies have systematically evaluated the clinical implications of admission hypokalemia in blunt liver trauma. The present study aims to assess whether serum potassium concentration upon admission correlates with injury severity, in-hospital morbidity, mortality, and length of hospital stay in patients with blunt hepatic trauma. We also explore its potential role as an accessible and cost-effective prognostic biomarker in the initial assessment and management of these patients.

## 2. Study Setting

This study was conducted at Policlinico Umberto I, a large tertiary academic hospital affiliated with Sapienza University of Rome. The institution is a designated Level I trauma center, serving a catchment area of more than two million residents and managing approximately 20% of the city’s major trauma cases annually.

### 2.1. Study Design and Population

We performed a retrospective observational cohort study including all patients admitted to our Emergency Department between January 2016 and December 2023 with radiologically confirmed blunt liver trauma. There were no age restrictions. Exclusion criteria included the following: penetrating abdominal trauma, isolated extra-abdominal injuries, or incomplete clinical and laboratory records.

### 2.2. Data Collection

The data were extracted from electronic medical records and institutional trauma registries. The following variables were collected:

- Demographics: age, sex;
- Mechanism of injury: road traffic collision, fall, or blunt assault;
- Physiological parameters at admission: systolic and diastolic blood pressure, heart rate, respiratory rate, Glasgow Coma Scale (GCS), and oxygen saturation;
- Laboratory findings: serum potassium, sodium, chloride, and creatinine;
- Imaging findings: abdominal CT scan;
- Trauma severity scores: Injury Severity Score (ISS), American Association for the Surgery of Trauma (AAST) liver injury grade.

### 2.3. Management Categories

Patients were classified into three groups based on the initial treatment strategy:

1. Non-operative management (NOM);
2. NOM with interventional radiology (IR) (e.g., angioembolization or percutaneous drainage);
3. Operative management, including perihepatic packing, total vascular hepatic exclusion (TVHE), or hepatic resection.

### 2.4. Outcomes and Definitions

The post-traumatic outcomes included the following:

- Morbidity, defined according to the Clavien–Dindo classification and the Centers for Disease Control and Prevention (CDC) criteria for surgical site infections (superficial, deep, or organ/space);
- Mortality, defined as in-hospital death or death within 30 days post-trauma;
- Length of stay (LOS), defined as the total number of hospital admission days.

### 2.5. Study Endpoints

The primary endpoint was to investigate the association between serum potassium concentration at admission and trauma severity, as measured by ISS and AAST grade. Secondary endpoints included correlations with in-hospital morbidity, mortality, and length of hospital stay.

### 2.6. Ethical Considerations

This study was conducted in accordance with the principles of the Declaration of Helsinki. Due to its retrospective and anonymized design, approval from the Institutional Review Board was waived. Written informed consent for clinical treatment and the scientific use of data was obtained from all patients at the time of hospital admission.

### 2.7. Statistical Analysis

The statistical analysis was conducted using MedCalc for Windows, version 14.0 (MedCalc Software, Mariakerke, Belgium).

- Continuous variables were expressed as mean ± standard deviation (SD) and compared using the Student’s *t*-test or ANOVA, as appropriate.
- Categorical variables were reported as frequencies and percentages and compared using the Chi-square or Fisher’s exact test.

Pearson’s or Spearman’s correlation coefficients were used to explore the relationships between potassium levels and continuous clinical variables, depending on normality (assessed by the Shapiro–Wilk test).

Multivariate linear and logistic regression models were constructed to identify independent predictors of serum potassium concentration and clinical outcomes. Multicollinearity was evaluated using the Variance Inflation Factor (VIF), ensuring that all predictors had a VIF < 2.

Variables with *p* < 0.05 in univariate analysis were entered into the multivariate models.

### 2.8. Patient Characteristics

The cohort included 164 patients with blunt hepatic trauma. The mean age was 38.3 years (range: 7–97), and 120 patients (73.2%) were male. The average Injury Severity Score (ISS) was 26.6 (range: 1–75). Additional patient details are summarized in Table 1.

### 2.9. Management Strategies

The severity of liver injury was graded according to the American Association for the Surgery of Trauma (AAST) scale:

- Grade I: 29 patients (17.7%);
- Grade II: 35 patients (21.3%);
- Grade III: 52 patients (31.7%);
- Grade IV: 29 patients (17.7%);
- Grade V: 19 patients (11.6%).

The initial management strategy was as follows:

- 78 patients (47.5%) were treated with non-operative management (NOM);
- 22 patients (13.4%) received NOM in combination with interventional radiology (IR);
- 64 patients (39.1%) underwent surgical treatment.

Polytrauma was present in 59 patients (35.9%), while 105 patients (64.1%) had isolated liver injuries.

#### 2.9.1. Definition of Management Outcomes

- Successful NOM was defined as clinical and hemodynamic stability without the need for delayed surgical intervention, with a resolution of symptoms and/or radiologic control.
- Failure of NOM was defined as any subsequent requirement for surgery due to hemodynamic deterioration, clinical worsening, or complications unresponsive to non-surgical measures.

#### 2.9.2. Successful Non-Operative Management

Among the 78 patients managed non-operatively, 56 (34.1%) were treated successfully with observation and supportive care, without any need for invasive procedures.

#### 2.9.3. NOM with Interventional Radiology

A total of 22 patients (13.4%) underwent NOM combined with minimally invasive radiological interventions:

- Angioembolization for active bleeding: 16 patients;
- Percutaneous drainage for intra-abdominal collections or bilomas: 6 patients.

Among these, 4 patients (18.2%) eventually required surgical intervention due to treatment failure.

### 2.10. Surgical Management

Sixty-four patients (39.1%) underwent operative treatment. The surgical indications were categorized as follows:

- Primary surgery due to hemodynamic instability at presentation: 38 patients;
- Secondary surgery following NOM failure: 26 patients (including 22 failures after NOM alone and 4 after NOM + IR).

### 2.11. Types of Surgical Procedures (n = 64)

The following surgical approaches were performed:

- Perihepatic packing and drainage for hemorrhage control: 38 patients (59.3%);
- Segmental or non-anatomic hepatic resection: 10 patients (15.6%);
- Splenectomy (due to concomitant splenic injury): 7 patients (10.9%);
- Bowel repair for perforation or injury: 5 patients (7.8%);
- Other abdominal procedures (e.g., cholecystectomy, mesenteric vessel ligation): 4 patients (6.2%).

### 2.12. Failure of Non-Operative Management

A total of 26 patients (33.3% of the 78 initially managed with NOM) experienced treatment failure and required surgery. These included the following:

- 22 failures after NOM alone (28.2%);
- 4 failures after NOM + IR (18.2%).

The following were the most common reasons for NOM failure:

- Secondary uncontrolled hemorrhage requiring surgical control (n = 13);
- Clinical deterioration with peritoneal signs suggestive of acute abdomen (n = 8);
- Persistent intra-abdominal abscesses not responsive to drainage (n = 3);
- Biliary leakage leading to bile peritonitis (n = 2).

A detailed summary of all management strategies and outcomes is provided in Table 2.

The mean length of hospital stay (LOS) for the entire cohort was 18.1 days (range: 1–98 days).

A total of 64 patients (39.0%) experienced trauma-related complications, with variable severity and clinical impact. The following were most common postoperative complications (Table 3):

- Intra-abdominal abscesses: 18 patients (28%);
- Wound infections: 12 patients (19%);
- Pulmonary complications (e.g., pneumonia, atelectasis): 10 patients (16%);
- Sepsis: 9 patients (14%);
- Biliary leaks or bilomas: 6 patients (9%);
- Hemorrhagic complications requiring transfusion or reoperation: 5 patients (8%);
- Enteric fistula or anastomotic leakage: 4 patients (6%).

In total, 33 patients (20.1%) died during hospitalization or shortly after admission. The main causes of death included the following:

- Exsanguination due to uncontrolled hemorrhage;
- Severe traumatic brain injury;
- Multiorgan failure.

Mortality was found to be significantly associated with the following:

- A higher Injury Severity Score (ISS);
- Hemodynamic instability upon admission;
- Early complications such as septic shock or refractory hemorrhage.

**Table 3:** Postoperative complications in patients with blunt hepatic injury.

Complication	Number of Patients (n)	Percentage (%)
Intra-abdominal abscesses	18	28%
Wound infections	12	19%
Pulmonary complications (pneumonia, atelectasis)	10	16%
Sepsis	9	14%
Biliary leaks or bilomas	6	9%
Hemorrhagic complications	5	8%
Enteric fistula or anastomotic leakage	4	6%

### 2.13. Potassium Levels at Admission

The admission serum potassium levels ranged from 2.2 to 6.0 mEq/L, based on standard clinical thresholds:

- Normokalemia (3.6–5.2 mEq/L) was observed in 96 patients (58.5%);
- Hypokalemia (<3.6 mEq/L) was found in 64 patients (39.1%);
- Hyperkalemia (>5.2 mEq/L) was documented in only 4 patients (2.4%).

**Figure 1:**
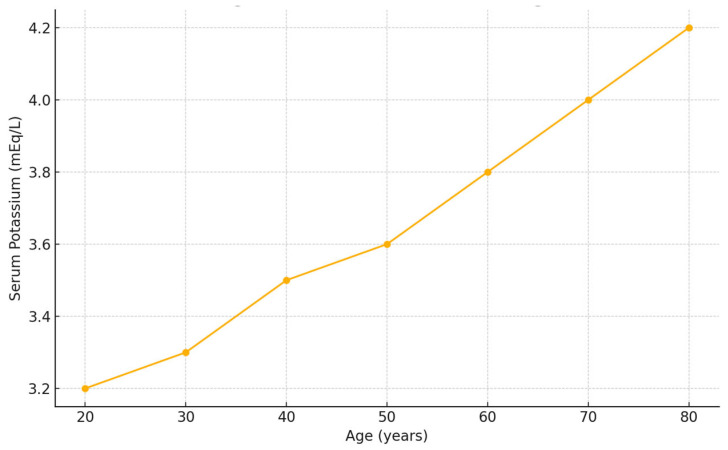
Correlation between serum potassium concentration and patient age in patients with blunt hepatic trauma. A significant positive correlation was observed between age and serum potassium concentration at admission (Pearson’s r = 0.24, *p* = 0.006).

A significant positive correlation was identified between serum potassium levels and patient age (*p* = 0.006), indicating that older patients tended to present with higher potassium values. This correlation was confirmed using Pearson’s test, as detailed in the statistical methods section (Figure 1).

#### 2.13.1. Univariate Analysis

A significant inverse correlation was found between serum potassium levels and liver trauma severity, as graded by the American Association for the Surgery of Trauma (AAST). Mean potassium levels progressively decreased with increasing AAST grades:

- AAST I: 3.871 mEq/L;
- AAST II: 3.841 mEq/L;
- AAST III: 3.827 mEq/L;
- AAST IV: 3.414 mEq/L;
- AAST V: 3.391 mEq/L.

**Figure 2:**
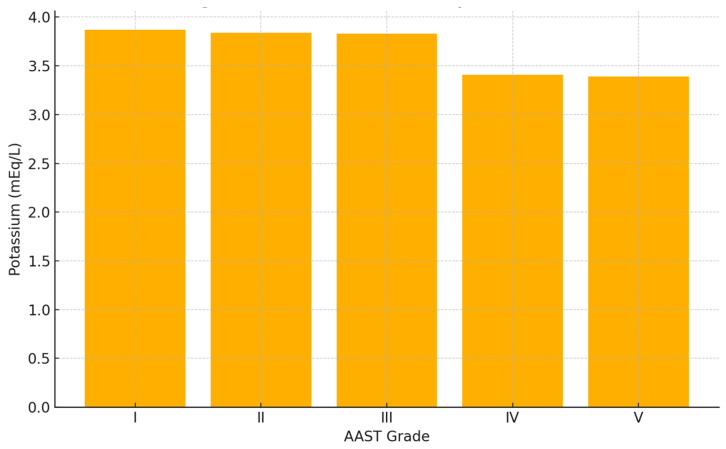
Mean serum potassium concentration by AAST liver injury grade in patients with blunt hepatic trauma. A significant inverse association was observed between AAST grade and potassium levels at admission (*p* = 0.01, ANOVA). Patient distribution: I (n = 29), II (n = 35), III (n = 52), IV (n = 29), V (n = 19).

This trend was statistically significant (*p* = 0.01) and is illustrated in Figure 2.

**Figure 3:**
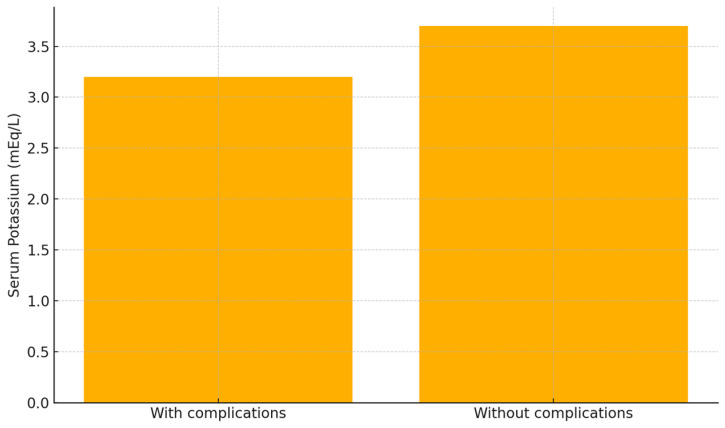
Comparison of serum potassium levels in patients with and without post-traumatic complications. Patients who developed complications (n = 64) had significantly lower potassium levels than those without complications (n = 100); mean values: 3.2 vs. 3.7 mEq/L (*p* = 0.0459, Student’s *t*-test).

Furthermore, patients who developed post-traumatic complications had significantly lower serum potassium levels at admission compared to those who did not (3.2 vs. 3.7 mEq/L; *p* = 0.0459) (Figure 3).

**Figure 4:**
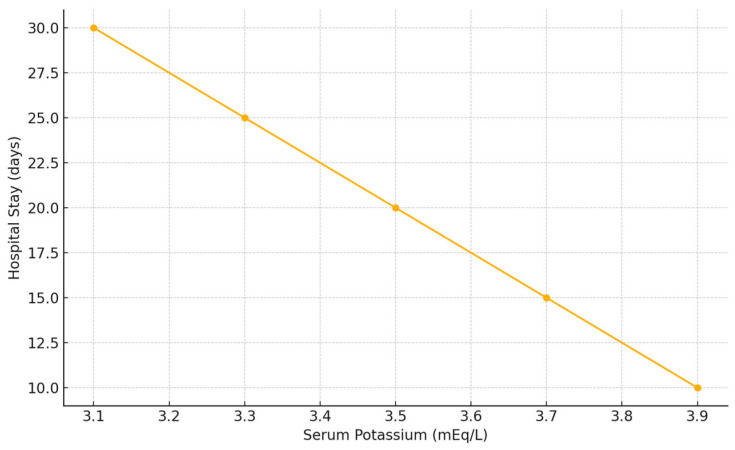
Correlation between serum potassium concentration and hospital length of stay in patients with blunt hepatic trauma. Lower potassium levels at admission were associated with longer hospital stay (Spearman’s rho = −0.21, *p* = 0.022).

In addition, length of hospital stay (LOS) was significantly longer in patients presenting with lower potassium concentrations (*p* = 0.022) (Figure 4).

No statistically significant correlation was observed between admission potassium levels and in-hospital mortality (*p* = not significant).

#### 2.13.2. Multivariate Analysis of Potassium Predictors

A multivariate linear regression analysis was performed to identify independent predictors of serum potassium concentration at admission (Table 4). Among the clinical and laboratory variables included, only the following were found to be significantly associated with serum potassium levels:

- Age (*p* = 0.0362);
- AAST liver injury grade (*p* = 0.0144).

Other variables—including creatinine, Injury Severity Score (ISS), gender, and presence of polytrauma—did not demonstrate statistical significance in the model.

These findings suggest that older age and more severe liver injury are independently correlated with higher or lower potassium values, respectively, at the time of hospital admission.

**Table 4:** Predictors of serum potassium levels (multivariate analysis).

Variable	Coefficient	Std. Error	t	*p*-Value
AAST	−0.1087	0.0439	−2.476	0.0144
Creatinine	0.1866	0.1495	1.248	0.2140
Age	0.0057	0.0027	2.114	0.0362
ISS	0.00001	0.0037	0.0025	0.9980
Gender	−0.0024	0.1203	−0.020	0.9840
Polytrauma	−0.1765	0.1189	−1.484	0.1399

ANOVA: *p* = 0.0113, F-ratio = 2.869.

#### 2.13.3. Multivariate Analysis of Morbidity

A separate multivariate logistic regression model was constructed to evaluate whether serum potassium concentration was independently associated with in-hospital morbidity (Table 5). In this analysis, potassium levels were not found to be an independent predictor of morbidity (*p* = 0.9995).

However, two variables were significantly associated with morbidity:

- Age, showing a borderline association (*p* = 0.0581);
- Injury Severity Score (ISS), which was strongly associated with increased morbidity (*p* < 0.0001).

These findings suggest that trauma severity, as reflected by ISS, remains the most robust predictor of postoperative complications in patients with blunt liver trauma. While potassium levels showed significant associations in univariate analysis, they did not retain independent prognostic value when controlling for other variables.

**Figure 5:**
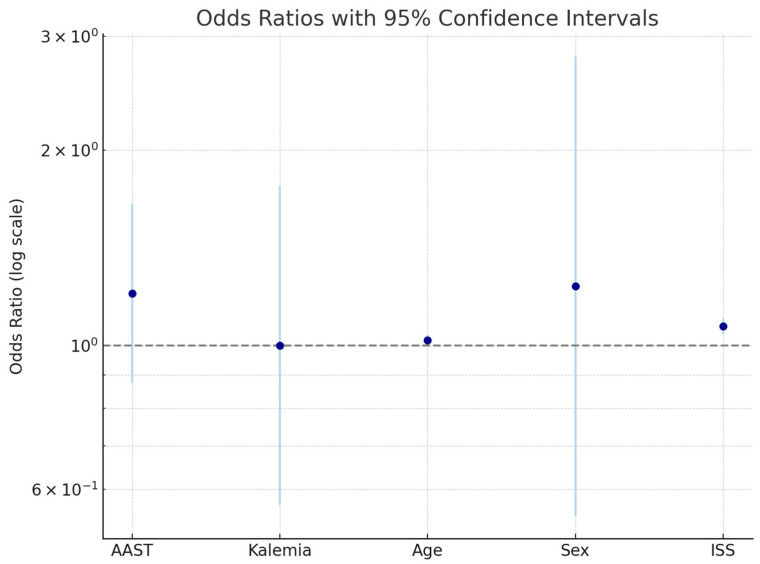
Odds ratios and 95% confidence intervals for clinical predictors of in-hospital morbidity in blunt hepatic trauma.

Figure 5 illustrates the odds ratios (ORs) and 95% confidence intervals (CIs) for the variables included in the multivariate logistic regression model evaluating predictors of in-hospital morbidity. An OR > 1 (above the dashed line at OR = 1) indicates an increased likelihood of complications, whereas an OR < 1 suggests a potential protective effect.

The following variables were plotted:

- AAST liver injury grade;
- Serum potassium concentration;
- Age;
- Sex;
- Injury Severity Score (ISS).

This visual summary supports the statistical findings that the ISS is the strongest predictor of morbidity, while serum potassium does not independently influence outcomes.

## 3. Discussion

Liver trauma is among the most common abdominal injuries encountered in trauma care. Most patients present with low-grade lesions, typically classified as World Society of Emergency Surgery (WSES) grades I–III or AAST-OIS grades I–III. Morbidity and mortality in hepatic trauma are primarily determined by injury severity, although numerous other factors—including hemodynamic instability, need for massive transfusion, and associated injuries—can significantly influence outcomes. High-grade liver trauma (AAST grades IV–V) is associated with mortality rates as high as 40–50% [9], although mortality is often multifactorial.

Notably, hepatic trauma rarely occurs in isolation. It is frequently accompanied by other major injuries—particularly to the head and thorax—which can independently contribute to poor outcomes. For this reason, it is important to differentiate liver-related morbidity from that related to associated injuries in order to accurately interpret clinical trajectories and management strategies.

Within this context, several studies have documented a post-traumatic reduction in serum potassium levels, most notably in liver trauma. This phenomenon is not explained by decreased intake or renal loss but rather by a redistribution of potassium into cells, mediated by adrenergic stimulation [9,10,11]. Beal et al. reported elevated plasma epinephrine levels in trauma patients with admission hypokalemia, with normalization of potassium values occurring within 24–36 h as catecholamine levels declined [4].

In the liver specifically, β_2_-adrenergic stimulation promotes potassium uptake both directly—via enhanced Na^+^/K^+^-ATPase activity—and indirectly, through glycogenolysis-induced hyperglycemia and subsequent insulin release [4,5,6,12,13]. These mechanisms act synergistically to lower extracellular potassium levels in the acute phase after trauma.

Pollice et al. were among the first to document this in liver trauma patients, showing persistent hypokalemia up to 7 days post-trauma despite adequate resuscitation [9]. However, their data were limited to a small prospective cohort (n = 11). Larger studies evaluating the clinical implications of this electrolyte disturbance have remained scarce.

Our study aimed to address this gap by investigating whether admission hypokalemia correlates with trauma severity and patient outcomes in a larger cohort. We found a significant inverse relationship between serum potassium and AAST grade, supporting the hypothesis that adrenergic activation in severe liver trauma contributes to potassium shifts. Additionally, hypokalemia was associated with a prolonged hospital stay and greater in-hospital morbidity, although it did not emerge as an independent predictor in multivariate models.

These results suggest that hypokalemia may reflect the physiological response to trauma severity, rather than directly driving outcomes. Nevertheless, it could serve as a useful surrogate marker for early clinical risk stratification in patients with blunt liver trauma.

The clinical significance of trauma-related hypokalemia has also been observed in other injury contexts. For example, a retrospective analysis of 520 patients with traumatic brain injury showed that hypokalemia (<3.5 mEq/L) at admission was associated with an increased need for emergency craniotomy (OR = 5.25; 95% CI: 2.06–13.40; *p* < 0.001) and a trend toward higher mortality [13]. This supports the broader concept of adrenergic-driven potassium redistribution as a systemic marker of trauma burden.

To our knowledge, this is the first study to demonstrate a statistically significant relationship between admission potassium levels and liver trauma severity in a large patient cohort. However, due to its retrospective design and potential confounding factors (e.g., timing of blood sampling, use of β-agonists, and fluid resuscitation protocols), the findings should be interpreted with caution.

Future prospective studies with serial potassium and catecholamine measurements are warranted to clarify the mechanistic pathways involved. Additionally, an exploration of whether modulation of the adrenergic response could impact outcomes may open new therapeutic avenues in trauma resuscitation.

## 4. Conclusions

This study suggests a potential inverse association between serum potassium levels at admission and the severity of blunt hepatic trauma, as well as relevant patient outcomes. In our cohort, lower potassium levels were observed in patients with higher AAST grades and were associated with increased in-hospital morbidity and longer hospital stays.

Given that serum potassium is a routinely measured, cost-effective laboratory parameter, it may serve as a useful adjunct for early risk stratification in patients presenting with blunt liver injury. Incorporating potassium levels into initial trauma assessments may help clinicians identify individuals at greater risk for adverse outcomes and guide timely, individualized interventions.

However, these findings must be interpreted with caution. The retrospective design, the absence of serial potassium monitoring, and the lack of control for potential confounders—including resuscitation protocols, medication use (e.g., beta-agonists), and timing of blood sampling—limit the ability to infer causality.

Further prospective studies with larger cohorts and standardized protocols are needed to validate our results and to explore the temporal relationship between potassium dynamics and clinical outcomes. Investigating the mechanistic interplay between adrenergic activation and electrolyte shifts may also provide a path toward targeted resuscitative strategies in trauma care.

## Figures and Tables

**Table 1:** Patient characteristics.

Variable	Value
Age (mean, range)	38.4 (7–97)
Gender	
Male	120
Female	44
ISS (mean, range)	26.7 (1–75)
AAST	
I	29
II	35
III	52
IV	29
V	19
Polytrauma	
No	105
Yes	59
Trauma Management	
Non-operative management	78
Operative management	64
Radiological intervention	22

**Table 2:** Management strategies and outcomes.

Management Strategy	Number of Patients (n)	Percentage (%)	Notes
Successful NOM	56	34.1%	No further intervention required
NOM + Interventional Radiology	22	13.4%	16 embolizations, 6 drainages; 4 failures (18.2%)
NOM Failure	26	33.3% of NOM	22 after NOM alone, 4 after NOM + IR
Surgical Treatment	64	39.1%	38 primary surgery, 26 NOM failures

**Table 5:** Predictors of morbidity (multivariate logistic regression).

Variable	Coefficient	Std. Error	*p*-Value	Odds Ratio	95% CI
AAST	0.1853	0.1626	0.2545	1.2036	0.8751–1.6554
Kalemia	0.0002	0.2892	0.9995	1.0002	0.5674–1.7631
Age	0.0185	0.0098	0.0581	1.0187	0.9994–1.0383
Sex	0.2104	0.4161	0.6132	1.2341	0.5460–2.7895
ISS	0.0686	0.0140	<0.0001	1.0710	1.0419–1.1008

## Data Availability

The original contributions presented in this study are included in the article. Further inquiries can be directed to the corresponding author.
